# Face and content validity of a novel, web-based otoscopy simulator for medical education

**DOI:** 10.1186/s40463-015-0060-z

**Published:** 2015-02-24

**Authors:** Brandon Wickens, Jordan Lewis, David P Morris, Murad Husein, Hanif M Ladak, Sumit K Agrawal

**Affiliations:** Department of Otolaryngology – Head and Neck Surgery, Western University, London, Ontario Canada; Department of Medical Biophysics, Western University, London, Ontario Canada; Department of Electrical & Computer Engineering, Western University, London, Ontario Canada; Division of Otolaryngology – Head and Neck Surgery, Department of Surgery, Dalhousie University, Halifax, Nova Scotia Canada; London Health Sciences Centre, Room B1-333, University Hospital, 339 Windermere Rd., London, Ontario N6A 5A5 Canada

**Keywords:** Otology, Otoscopy, Medical education, Simulation, Web-based, Simulator, Otitis media, Otitis media with effusion, Metrics, Evaluation

## Abstract

**Background:**

Despite the fact that otoscopy is a widely used and taught diagnostic tool during medical training, errors in diagnosis are common. Physical otoscopy simulators have high fidelity, but they can be expensive and only a limited number of students can use them at a given time.

**Objectives:**

1) To develop a purely web-based otoscopy simulator that can easily be distributed to students over the internet. 2) To assess face and content validity of the simulator by surveying experts in otoscopy.

**Methods:**

An otoscopy simulator, OtoTrain™, was developed at Western University using web-based programming and Unity 3D. Eleven experts from academic institutions in North America were recruited to test the simulator and respond to an online questionnaire. A 7-point Likert scale was used to answer questions related to face validity (realism of the simulator), content validity (expert evaluation of subject matter and test items), and applicability to medical training.

**Results:**

The mean responses for the face validity, content validity, and applicability to medical training portions of the questionnaire were all ≤3, falling between the “Agree”, “Mostly Agree”, and “Strongly Agree” categories. The responses suggest good face and content validity of the simulator. Open-ended questions revealed that the primary drawbacks of the simulator were the lack of a haptic arm for force feedback, a need for increased focus on pneumatic otoscopy, and few rare disorders shown on otoscopy.

**Conclusion:**

OtoTrain™ is a novel, web-based otoscopy simulator that can be easily distributed and used by students on a variety of platforms. Initial face and content validity was encouraging, and a skills transference study is planned following further modifications and improvements to the simulator.

## Introduction

Otologic diseases are highly prevalent, can significantly impair function, and are a costly burden to the healthcare system. Otitis media with effusion has affected 2.1% of the global population, and it can cause deficits in language acquisition and social interaction [1]. Acute otitis media is the most common reason antibiotics are prescribed for children in the United States [2]. Expertise in otoscopy is essential for assessing and diagnosing these illnesses, however studies have demonstrated the need for improved otoscopy education. In 1992, Fisher and Pfleiderer revealed that general practitioners possessed comparable otoscopy skills to medical students, thereby demonstrating room for improvement in otoscopy training [3]. Studies evaluating pediatric residents’ assessment of ear disease determined a 41% accuracy rate and only a slight agreement between resident diagnosis and tympanometry [4,5]. Pichichero and Poole evaluated the ability of General Practitioners and Pediatricians to diagnose otitis media with effusion and acute otitis media, and their diagnostic accuracy rates were similar ranging from 36-51% [6,7].

Otoscopy simulators to date have primarily relied on physical models of the entire head, or of the ear in isolation [8-10]. These simulators have the advantage of high fidelity, as trainees use an actual otoscope to navigate through the synthetic ear and visualize the images of the tympanic membrane. However, physical simulators can be costly, they require dedicated space within the educational institution, they are ‘single-user’ making scheduling numerous trainees tedious, and distribution to developing nations may be difficult.

Recently, computer-based technology and web-based technology in medical education have been gaining in popularity [11-13]. Pure internet-based simulators are relatively low-cost in terms of servers and bandwidth, and they allow hundreds of students to use the platform simultaneously. Students can access the simulators on any internet-connected computer or mobile device, so they are not limited to their educational institution or particular scheduled times. Administrators and educators can also benefit with the ability to manage classes online, send reminder e-mails with links to the software, and track students’ progress and results remotely. Achieving realism in computer simulators can be difficult, therefore the use of haptic devices for force feedback or hybrid-simulators may be necessary.

The primary objective of this study was to develop a novel web-based otoscopy simulator that could easily and inexpensively be deployed to any students with an internet connection. The secondary objective was to survey experts to establish face and content validity of the simulator.

## Methods

### Simulator development

The otoscopy simulator was designed and developed at Western University in the Auditory Biophysics Laboratory and is called OtoTrain. Models of the auricle and ear canal were created in Unity 3D (Unity Technologies, San Francisco, California), a gaming engine that works across desktop, mobile, web, and console-based platforms. The Unity plug-in allowed for real-time interaction with the 3D models of the ear over the internet.

Users can log into the system as either an Administrator, Examiner, or Trainee. The Administrator module provides the ability to manage entire classes and e-mail addresses, create/delete accounts, and manage the program settings. The Examiner module allows the user to create custom exams with multiple-choice questions and a choice of pathologies. The latest version of the simulator allows examiners to upload tympanic membrane images from their personal collections, and these are dynamically attached to the 3D ear canal models within the simulator. Examiners can share their exams with other users, place them into ‘study’ mode (allows students to browse pathologies and receive the answers instantly), or place them into a formal ‘exam’ mode (students take an exam and the results are sent to the examiner). Examiners have the ability to send a link of their exam to an entire class, set time limits for completion, track which students have completed the exam, and automatically tabulate results.

**Figure 1:**
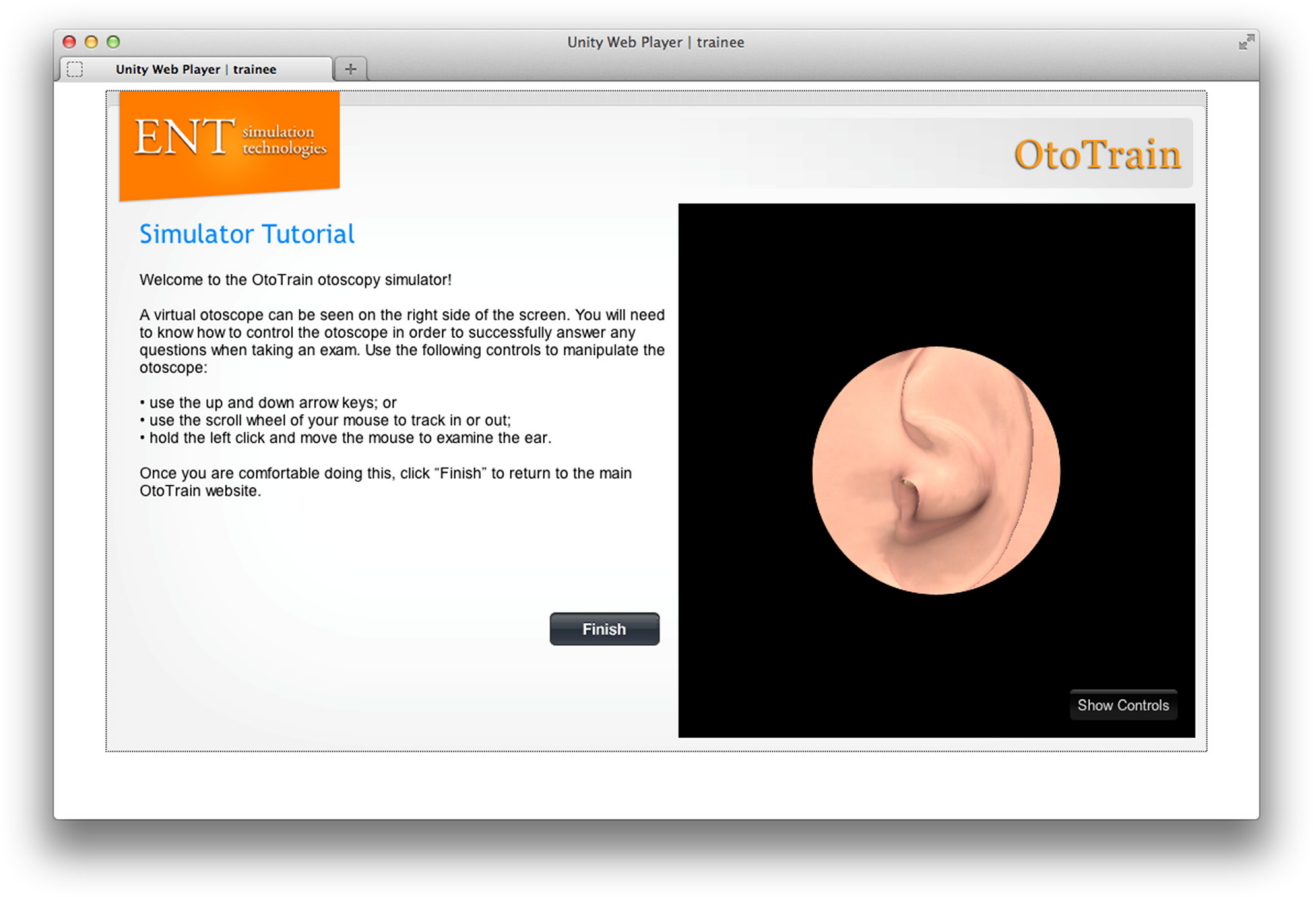
**Otoscopy simulator tutorial module.**

**Figure 2:**
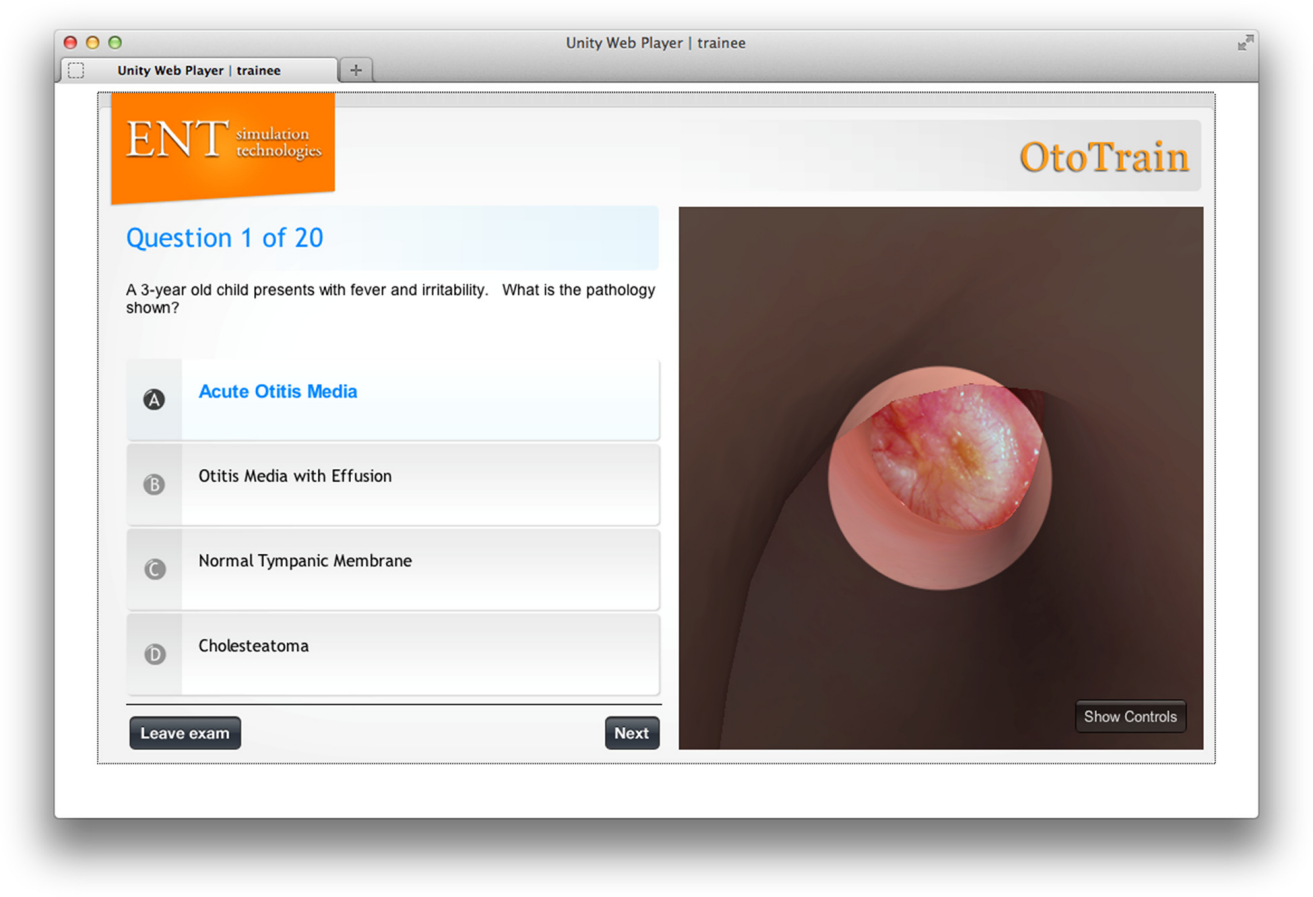
**Otoscopy module demonstrating acute otitis media.** LEFT PANEL - Examiners can create their own clinical scenarios and multiple-choice questions. RIGHT PANEL - The virtual speculum can be made translucent in 'study mode' as shown, or opaque in 'test mode' to increase difficulty.

**Figure 3:**
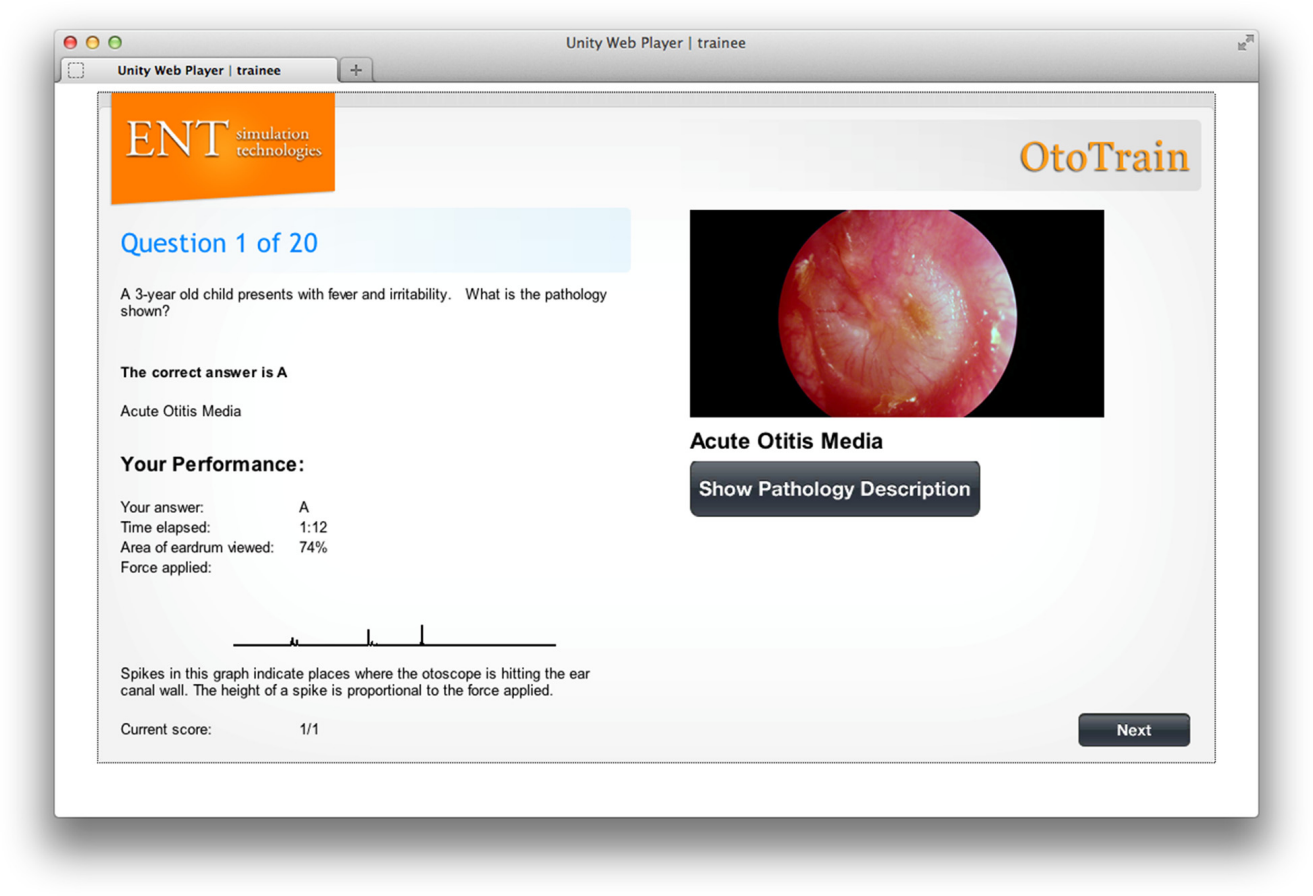
**Performance feedback following module completion.**

**Figure 4:**
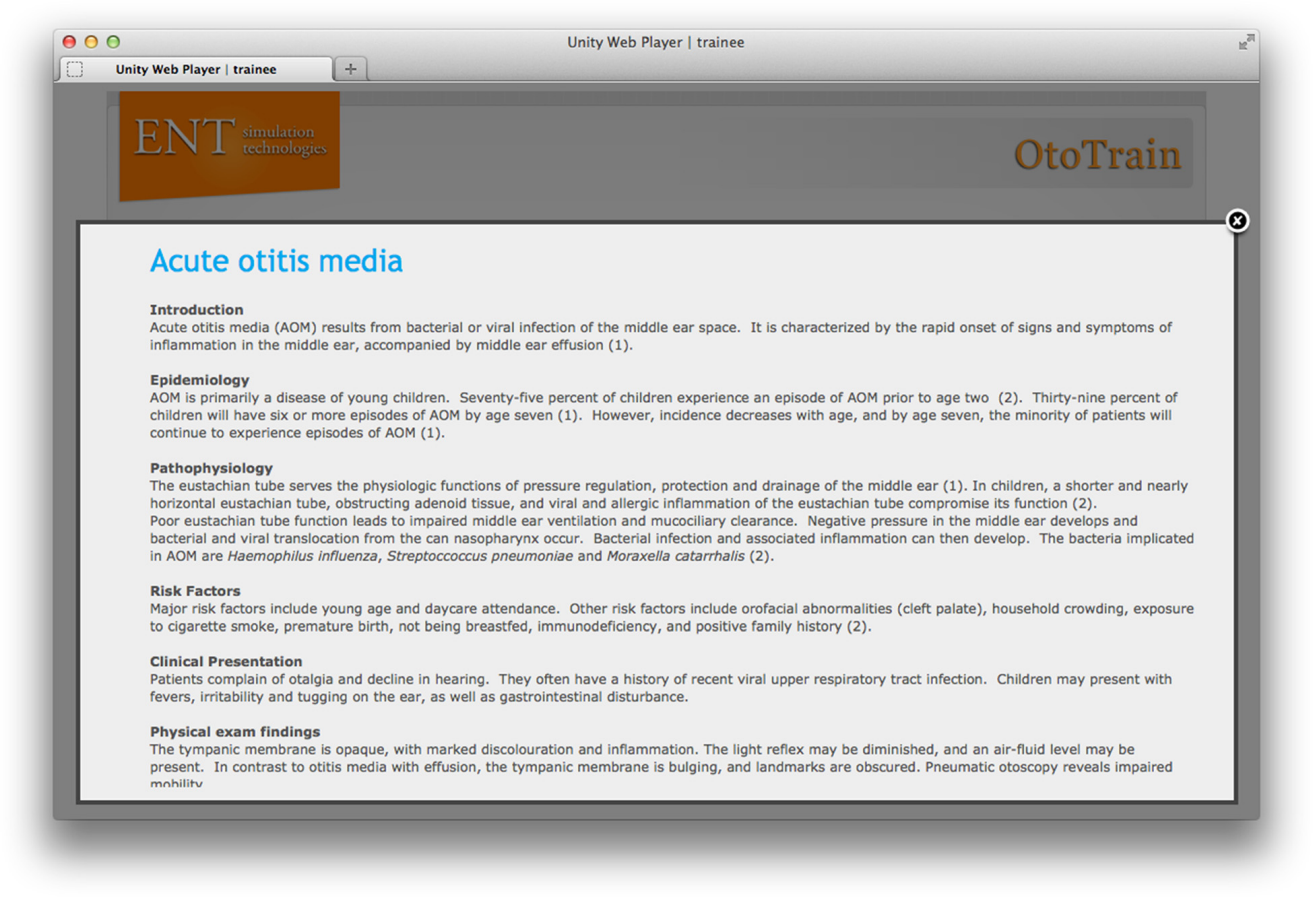
**Pathology descriptions can be accessed following each question in study mode.**

**Figure 5:**
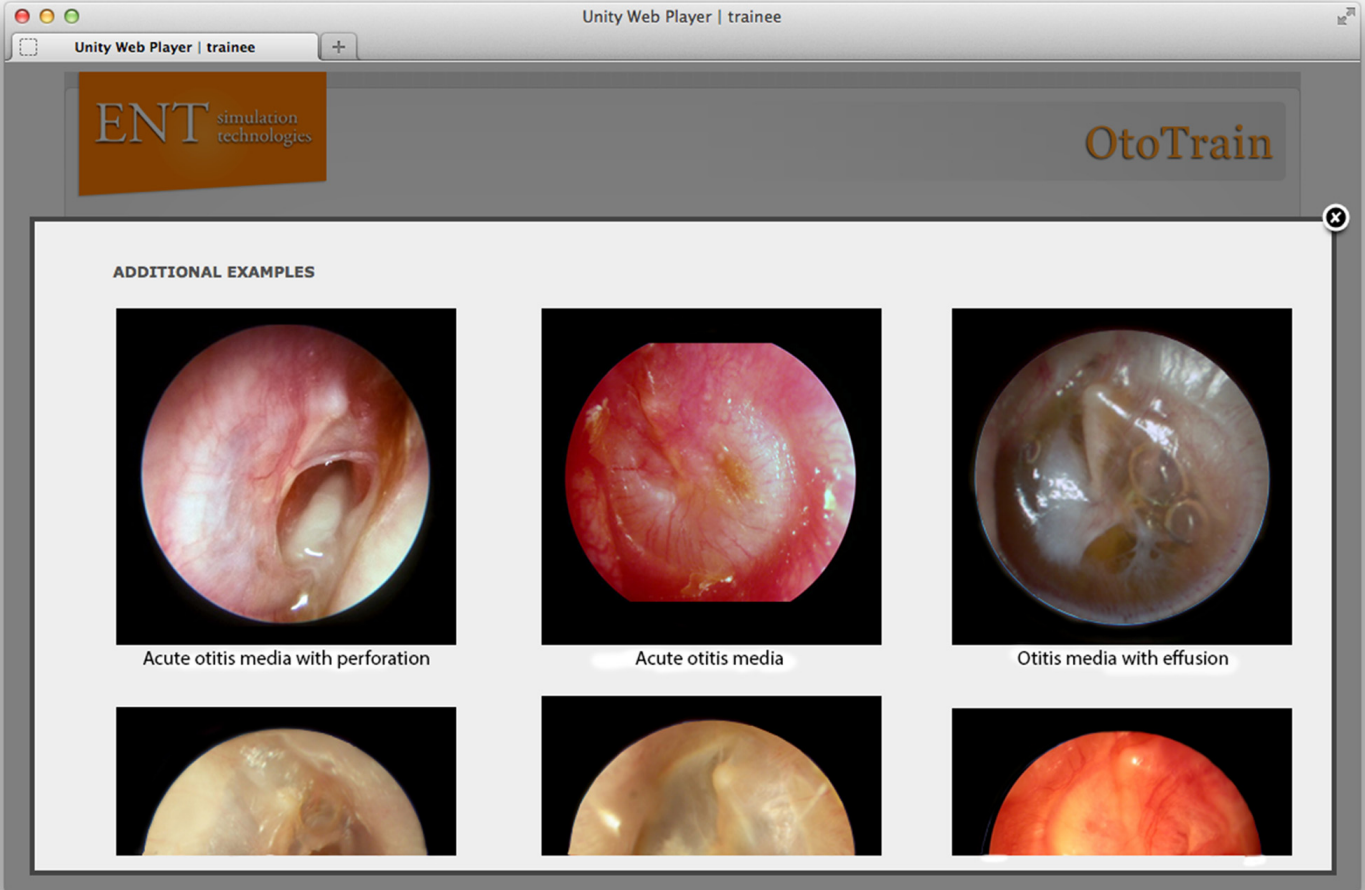
**Additional images and examples are shown for each pathology.**

Trainees can login to the simulator and learn otoscopic techniques, browse pathologies, or take a simulator tutorial (Figure 1). When browsing in ‘study’ mode, the speculum is made translucent to ease the navigation down the ear canal and keep the students oriented (Figure 2). During the simulation, the virtual otoscopy is performed using a mouse/trackpad and keyboard. Feedback regarding the accuracy of the diagnosis and various metrics are immediately given when in ‘study’ mode (Figure 3). Current metrics include the percentage of tympanic membrane visualized, time to completion, and ‘force’ against the ear canal. The virtual ‘force’ is calculated in proportion to the displacement magnitude past the ear canal wall, and no actual haptic feedback is felt by the trainees. Trainees can then access further pathology descriptions and image examples to enhance their learning around each case (Figures 4 and 5).

### OtoTrain validation

A comprehensive face and content validity questionnaire was developed by the authors as a validated tool assessing otoscopy simulation could not be found in the literature. The survey contained three assessment components. The first used a 7-point Likert scale (1 – strongly agree, 4 – neutral, 7 – strongly disagree) to assess the face and content validity of the simulator, applicability to training curriculum, and overall usability. The second component compared the otoscopy simulator to traditional lecture and atlas methods of otoscopy education, to assess whether experts felt this novel simulator would be beneficial. The third component solicited qualitative feedback using open-text questions regarding perceived advantages or limitations of the simulator, alternative applications and suggestions for improvement of the OtoTrain™ prototype. The survey was entered into an online survey tool, Survey Monkey (www.surveymonkey.com, Palo Alto, California).

Eleven experts actively involved in teaching otoscopy were contacted to evaluate the simulator and complete the assessments. The experts were based in the following academic institutions: Stanford University, Ohio State University, Dalhousie University, McGill University, University of Ottawa, University of Toronto, University of British Columbia, and the National Centre of Audiology.

An initial video oriented the experts to the simulator, and they were then given an unlimited time with access to the Administrator, Examiner, and Trainee modules. The results of the survey were then collected electronically.

## Results

A total of eleven experts completed the survey. There were 7 males and 4 females, with a mean number of years in practice of 11.1 years (range, 1–18 years). By specialty, there were 5 Neurotologists, 5 Pediatric Otolaryngologists, and 1 Audiologist.

### Face validity

The degree to which the simulator appears to be realistic was assessed using a 7-point Likert scale ranging from 1 (strongly agree) to 7 (strongly disagree). Two domains were assessed: (i) appearance of the normal/abnormal external auditory canal anatomy, and (ii) appearance of the normal/abnormal tympanic membrane (eardrum) anatomy. The results are tabulated in Table 1. The mean scores across both study domains measured at ≤ 3, falling between the “Agree”, “Mostly Agree”, and “Strongly Agree” categories. This was considered to be an acceptable realistic representation of the relevant anatomy.

**Table 1:** **Survey results**

**Domain**	**Mean Likert scale ranking** ^**1**^ **(SD) (n = 11)**
**Face validity**	
Realistic representation of normal/abnormal external auditory canal anatomy	2.7 (1.5)
Realistic representation of normal/abnormal tympanic membrane anatomy	2.3 (1.3)
**Content validity**	
Teaches importance of assessing external auditory canal	2.4 (0.8)
Teaches importance of assessing entire tympanic membrane	2.2 (1.3)
Provides adequate breadth of pathologies	2.1 (1.4)
Is a useful introduction to otoscopy	1.5 (0.8)
**Applicability to medical training**	
Useful for training of medical students/audiology students	1.5 (0.8)
Useful for training of non-Otolaryngology residents (Family Medicine, Paediatrics, Emergency Medicine, etc.)	1.7 (1.0)
Useful for training of Otolaryngology residents	2.5 (1.3)
Recommend for use in continuing medical education	1.8 (1.0)
User-friendly	1.7 (1.1)

^1^Likert scale ranking – 1- strongly agree, 4 – neutral, 7 – strongly disagree.

### Content validity

The degree to which the simulator addresses all subject material and curriculum requirements was assessed using the same Likert scale across the following 3 domains: (i) assessment of the external auditory canal and tympanic membrane; (ii) exposure to adequate breadth of pathology; and (iii) utility as an introduction to otoscopy (Table 1). The mean scores across all three domains again measured ≤3, and were thus considered acceptable across all domains.

### Applicability to the medical training curriculum

The global rating of the simulator characterized the success of the simulator overall (Table 1). This included the experts' opinions. individually and collectively, as to whether the simulator serves as a useful training tool for uptake by medical/audiology students, non-Otolaryngology residents, and Otolaryngology residents. The global rating also examined ease of use of the simulator, and whether the simulator should be recommended for use in medical education. The calculated means determined from the statement parameters revealed consistently positive scores (≤3), and were considered acceptable across all domains.

### Comparison of training methods

Experts ranked whether they felt that the OtoTrain™ simulator was superior, equivalent, or inferior to the traditional atlas- or lecture-based method of otoscopy education. Four domains were assessed: (i) learning tympanic membrane anatomy; (ii) learning external auditory canal anatomy; (iii) learning manual otoscopic technique; and (iv) ease of access/availability of the training method (Table 2). In each domain, the simulator tool was deemed superior to the atlas/lecture-based method, with the exception of learning tympanic membrane anatomy in which an equal percentage of experts considered it to be superior or equivalent.

**Table 2:** **Comparison of training methods**

**Statement**	**Online simulator superior**	**Equivalent**	**Atlas or lecture superior**	**Unsure**
Learning tympanic membrane anatomy	**45.5%**	**45.5%**	0.0%	9.1%
Learning external auditory canal anatomy	**63.6%**	36.4%	0.0%	0.0%
Learning otoscopy technique	**63.6%**	27.3%	0.0%	9.1%
Availability of training technique	**54.5%**	9.1%	27.3%	9.1%

Items in bold indicate the highest percentage in each domain.

### Qualitative feedback

Although the means of all scores were ≤ 3, suggesting favourable responses to the otoscopy simulator overall, several helpful suggestions were derived from the qualitative component of the survey. The most common suggested improvements included:

1. Need for haptic feedback (by using a device, such as a haptic arm, to simulate controlling and navigating the otoscope into the ear canal)
2. Need for greater emphasis on importance of pneumatic otoscopy (visualizing movement of eardrum due to various pressure changes)
3. Need for including more eardrum pathologies, such as eardrums with hearing aid complications, post-surgical scars, and different skin pathologies.

Other suggested refinements included simultaneously displaying both the left and right ear in the pathology pictures, including more pictures and less text when describing how to conduct otoscopy, and incorporating pictures of less obvious pathologies as student skill improves.

## Discussion

The ability to perform accurate otoscopy is necessary for many medical and auditory professionals including Family Physicians, Pediatricians, Emergency Room Physicians, Nurses, and Audiologists. Despite its importance, diagnostic accuracy rates are low which necessitates improved teaching and experience in the early stages of training [4-7].

The results of the survey were encouraging with positive responses for face validity, content validity, and applicability to the medical training curriculum. Direct comparison against the traditional atlas/lecture method of teaching was also favourable for OtoTrain™.

The qualitative feedback section did highlight some limitations of a web-based solution for otoscopy simulation. The need for additional realism during the act of otoscopy was highlighted. This is a difficult problem to overcome when trainees are using inexpensive consumer hardware or mobile devices in order to access the simulator. One solution would be to incorporate an inexpensive haptic device to provide force feedback when navigating the otoscope, and this has already been programmed into the simulator. While this would be reasonable on a desktop or notebook computer, this would not work on portable/mobile devices. Another solution would be to develop an inexpensive hybrid model with a face ‘mask’ attached to a consumer monitor. The advantage of a hybrid solution is that the trainee can hold and manipulate a real otoscope, feel the interaction between the speculum and the ear canal, and practice how to position their hands. This has been previously accomplished in ophthalmoscopy simulators, such as the Eyesi Direct Opthalmoscope Simulator (VRmagic GmbH, Mannheim, Germany), however its realism and efficacy would need further study.

The lack of tympanic membrane pathologies has also been addressed in the next version of the simulator. The examiner now has the ability to upload any photos in their personal collection to the simulator. These photos are then fit onto the end of the 3D ear canal so that they can be displayed interactively.

Pneumatic otoscopy is challenging to accurately simulate in a virtual-reality simulator. Physical simulators have recently incorporated pneumatic otoscopy [8,9], however in a virtual-reality system it would have to be displayed as an animation between a static and a pressurized tympanic membrane. The pressurization deformation could be estimated and applied to the image if actual pneumatic images are not available.

Advantages of internet-based learning have been established [11-13], with the primary advantages in this case being lower cost and wide accessibility around the world. Internet-based simulators can be used by hundreds of students simultaneously on mobile platforms anywhere. Examiners can also manage large classes of students remotely allowing for a great deal of flexibility. Also, in addition to being able to add unlimited tympanic membrane pathologies, virtual-reality simulators have the advantage of unlimited external auditory canal sizes, shapes/curvatures, and pathologies. The external auditory canal and meatus are typically static in most physical simulators.

Limitations of this study include the lack of direct comparisons against existing physical simulators. Experts were asked to compare OtoTrain™ against their existing teaching paradigm, which consisted of lectures/atlas-based teaching. However, high-fidelity, low-accessibility physical simulators may be used in conjunction with low-fidelity, high-accessibility virtual-reality simulators to maximize exposure and availability for trainees.

Finally, face and content validity only represent the initial stages of validation for any simulator. Modifications and updates to OtoTrain™ have already occurred based on the feedback from this study, and research ethics board approval has already been received for a skills transference study at Western University over the upcoming year. This will determine if the use of OtoTrain™ actually improves the diagnostic accuracy of medical students.

## Conclusion

OtoTrain™ is a novel, web-based otoscopy simulator that can be easily distributed and used by students on a variety of platforms. Initial face and content validity was encouraging, and a skills transference study is planned following further modifications and improvements to the simulator.
